# The effect of on-site CT-derived fractional flow reserve on the management of decision making for patients with stable chest pain (TARGET trial): objective, rationale, and design

**DOI:** 10.1186/s13063-020-04649-9

**Published:** 2020-08-20

**Authors:** Junjie Yang, Dongkai Shan, Mei Dong, Zhiqiang Wang, Xiang Ma, Xinyang Hu, Hesong Zeng, Yundai Chen

**Affiliations:** 1 Department of Cardiology, Chinese PLA General Hospital, 28 Fuxing Rd, Haidian District, Beijing, 100853 People’s Republic of China; 2 Department of Cardiology, Qilu Hospital of Shandong University, Jinan, People’s Republic of China; 3 Department of Cardiology, Anzhen Hospital, Capital Medical University, Beijing, People’s Republic of China; 4 Department of Cardiology, First Affiliated Hospital of Xinjiang Medical University, Urumchi, People’s Republic of China; 5 Department of Cardiology, Second Affiliated Hospital, School of Medicine, Zhejiang University, Hangzhou, People’s Republic of China; 6 Department of Cardiology, Tongji Hospital, Tongji Medical College, Huazhong University of Science and Technology, Wuhan, People’s Republic of China

**Keywords:** Management, Coronary artery disease, Non-invasive test, Coronary computed tomographic angiography, Fractional flow reserve

## Abstract

**Background:**

The diagnostic accuracy of CT-derived fractional flow reserve (CT-FFR) in clinical application has been well validated. This advanced technology focus on evaluating anatomical stenosis and functional ischemia simultaneously. However, the effect of CT-FFR on the management of decision making has not been fully evaluated in randomized controlled design.

**Method/design:**

TARGET study is a pragmatic, multicenter, prospective, open-label, and randomized controlled trial evaluating the effect of a CCTA/CT-FFR strategy (group A) versus usual care (group B) on intermediate-to-high risk patients with suspected CAD who undergo clinically indicated diagnostic evaluation. A total sample size of 1216 subjects will be enrolled and followed up for 12 months. This study will be performed in 6 Chinese hospitals, and the primary endpoint is the planned ICA without significant obstructive CAD within 90 days. The secondary endpoints include MACE, quality of life, medical expenditure, and cumulative radiation exposure during 1-year follow-up.

**Discussion:**

The study will provide information to patients, health care providers, and other stakeholders in China about which strategy could be more effective in the management of intermediate-to-high risk patients with suspect CAD.

**Trial registration:**

ClinicalTrials.gov NCT03901326. Registered on 3 April 2019.

## Background

Coronary computed tomographic angiography (CCTA) has become an excellent rule-out test for suspect coronary artery disease (CAD). The recommendation for CCTA was even expanded by one national guideline as a first-line test to patients with intermediate and high likelihood of CAD based on their cost-effectiveness analysis suggesting that this would be a lower-cost strategy. However, the relative low specificity of CCTA in the diagnosis of functional myocardial ischemia makes it difficult to act as a real gatekeeper for the patients to be referred to invasive coronary angiography (ICA). In developing countries like China, for the patients with prior CCTA test who are subject to downstream ICA, over 30% of them were found to have no obstructive CAD [1]. Even more, the invasive procedure seems to be much more frequent when CCTA was introduced to clinical practice in some pragmatic clinical trials [2, 3].

Recently, CT-FFR, a kind of novel functional assessment derived from CCTA, dramatically increases the specificity of diagnosis on flow-limiting coronary stenosis, enabling CCTA to become a more robust non-invasive strategy. It showed a great potential in detecting functional myocardial ischemia related to coronary-specific lesion (in Discovery-Flow, DEFACTO, and NXT trials) [4–6]. Moreover, clinical care guided by CT-FFR could provide benefits with equivalent clinical outcomes and lower expenditure, compared with routine clinical care over 1-year follow-up (Platform trial) [7]. In addition, ADVANCE study revealed that CT-FFR modified the treatment recommendation which might reduce unnecessary ICA, predict revascularization, and further identify subjects at low risk of adverse events through 90 days [8].

However, these studies were not randomized designed and selection bias still existed. Also, the cost-effectiveness of CT-FFR in clinical practice remains to be determined, especially in developing countries. The purpose of this present study will be to evaluate whether CCTA/CT-FFR outperforms the usual care in ruling out patients without significantly obstructive CAD before invasive catheterization and improving clinical prognosis during follow-up in a randomized design.

## Method/design

### Study aim

TARGET trial is a multicenter, prospective, open-label, and pragmatic randomized controlled trial evaluating the effect of a CCTA/CT-FFR strategy (group A) on management decision making versus usual care (group B) in intermediate-to-high risk patients with suspected CAD who undergo clinically indicated diagnostic evaluation.

Recruitment commenced in August 2019. Additional file 1 is the Standard Protocol Items: Recommendations for Interventional Trials (SPIRIT) checklist. The schedule of enrollment and assessments follows the SPIRIT Figure. The study protocol (Version 2.0/201812) and other relevant documentations have been approved by the institutional human research ethic committee of Chinese PLA General Hospital and the relevant national ethics committees as well as registered on ClinicalTrials.gov identifier: NCT03901326.

### Setting

**Fig. 1:**
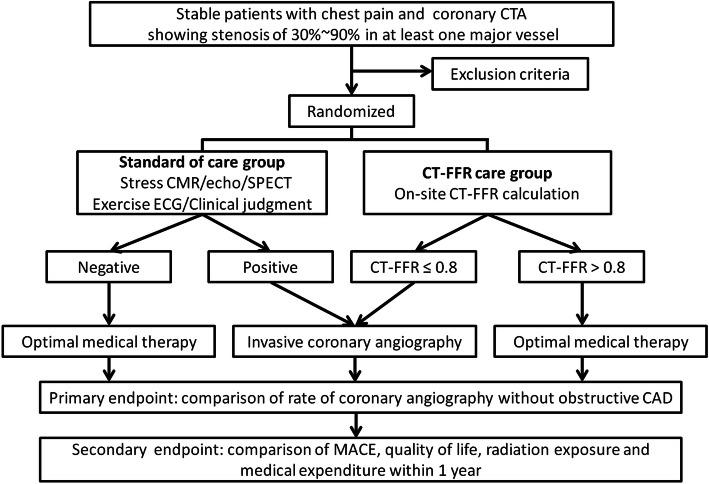
The flow chart of TARGET trial. To evaluate the effect of CT-FFR care strategy on improving decision making management, subjects enrolled will be randomized into either standard of care arm or CT-FFR care arm

This multicenter randomized controlled clinical trial will be carried out in 6 tertiary hospitals across China, all of which has the volume of over 200 patients in outpatient area of cardiology division each working day. Participating subjects will be enrolled and subsequently assigned to either usual care group or CT-FFR care group via computer-generated random numbers (1:1 ratio) (Fig. 1). The trial accords with the SPIRIT guidelines. Core lab has been established to receive all the imaging data for analysis, and two trained clinicians will conduct all of the measurements. The treatments (both intervention and control) will be delivered by licensed clinicians in the participating sites. Central telephone is used for allocation of sequence. Participants will be randomized to the CT-FFR examination group or usual care group using a randomization procedure. The cardiologist will be aware of patients’ group allocation because they will provide the trial intervention, but they will not be involved in the analysis. Participants are not blind to their group allocation, nor are their physicians who are informed of screening results of their patients (if the patient consents) and give the recommendation by outpatient or telephone. There will be no special criteria for discontinuing or modifying allocated interventions.

### Eligibility criteria

#### Inclusion criteria

Consecutive patients with new-onset chest pain suspicious for CAD will be included. Subjects with intermediate-to-high likelihood of CAD will be recruited based on various typicality of chest pain. Another major inclusion criterion is the CCTA result which showed that the diameter stenosis is between 30 and 90% in at least one major coronary artery (coronary artery diameter ≥ 2.5 mm).

The typicality of the chest pain were determined by three characteristics of chest pain, including central chest discomfort lasting below 15 min, provoked by exertion or emotional stress, and relieved by rest or nitrates. This definition is similar with the NICE guideline update (2016) [9]. Non-anginal pain was defined as the presence or absence of only one characteristic of chest pain. Atypical angina was defined as the presence of two characteristics. Typical angina was defined as the presence of all three characteristics above. For the mild coronary stenosis (30–49%), patients with typical angina will be recruited. For the intermediate stenosis (50–69%), patients with atypical angina or non-anginal pain will be recruited. For the severe stenosis (70–90%), only patients with non-anginal pain will be included.

Agreement to participate in this trial will be necessary and informed consents will be obtained from all subjects before recruiting.

#### Exclusion criteria

- Diagnosed or suspected acute coronary syndrome requiring hospitalization or emergent testing;
- Hemodynamically or clinically unstable condition systolic blood pressure < 90 mmHg or serious atrial or ventricular arrhythmias;
- Known CAD with prior myocardial infarction, percutaneous coronary intervention (PCI), coronary artery bypass graft (CABG), or any angiographic evidence of ≥ 50% stenosis in any major coronary artery;
- Patients with left main branch stenosis> 50% and/or 3-vessel disease;
- Known severe congenital, valvular (moderate and above), or cardiomyopathy process (hypertrophic cardiomyopathy or reduced systolic left ventricular function ≤ 40%) which could explain cardiac symptoms;
- Unable to provide written informed consent or participate in long-term follow-up.

### Measurement

**Fig. 2:**
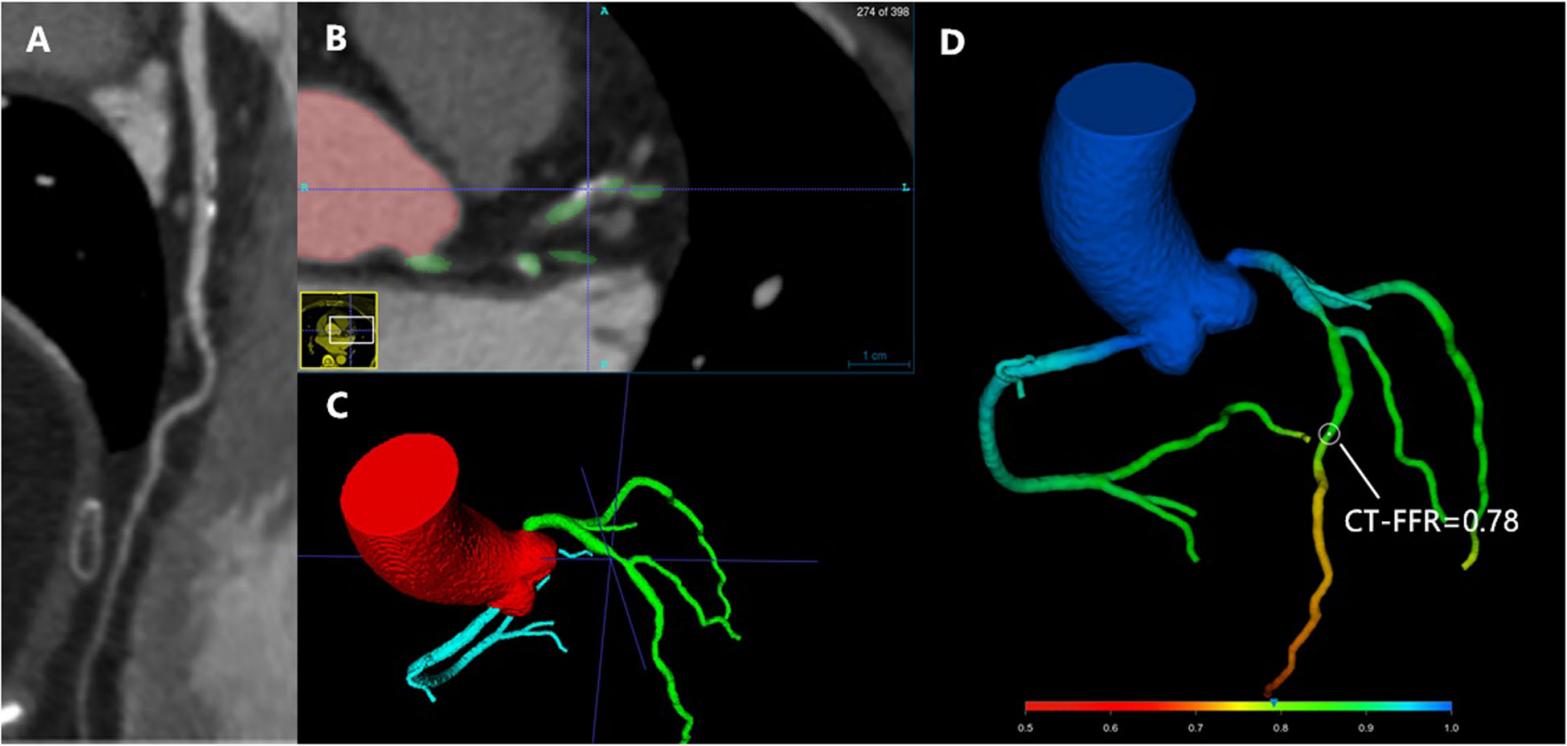
Schematic presentation of DeepFFR measurement on coronary artery stenosis. **a** Routine CCTA analysis was performed and a mixed plaque with 50% stenosis was found in the proximal left anterior descending (LAD). **b** Cross-sectional image of the lesion. **c** 3D coronary artery tree was generated in DeepFFR analysis module. **d** Lesion-specific FFR value derived from CT coronary artery tree was calculated and demonstrated. CT-FFR value is 0.78

CCTA image is obtained before the patient’s first visit and assessment. When subjects are randomized to the CCTA/CT-FFR arm, on-site FFR based on the CCTA imaging (DeepFFR V1.0.0, Beijing CuraCloud Technology Co., Ltd., Beijing, China) will be measured. DeepFFR workstation is very dedicated software utilizing the original CCTA imaging to meter simulated FFR values in artificial intelligence (AI) model, which has been introduced in previous article [10]. The calculation process could be summarized as follows: the first step is to extract a 3D coronary artery model and generate coronary centerlines which are similar to the routine reconstruction of CCTA. A modified 3D U-Net like model is employed to generate a major coronary artery tree followed by a graph cut to refine the boundary of the arteries. The centerlines are extracted using a minimal path extraction filter. Then, a novel path-based deep learning model, referred to DeepFFR, is used to predict the simulated FFR values on the vascular centerlines. Deep learning algorithm is used to establish characteristic sample database of coronary hemodynamic characteristic parameters. When the deep training model is proved to be valid, it is applied to a new lesion-specific measurement. DeepFFR system consists of a multi-layer perceptron network (MLP) and a bidirectional multi-layer recursive neural network (BRNN). The whole model can process variable-length input, and each point of the input sequence is transferred separately corresponding to MLP; the output of the MLP is transferred into the BRNN to optimize the sequence model. In comparison with the previous technology, the major advantage of DeepFFR model is more accurate because of the incorporation of context information on target FFR along the vessel path. More specifically, DeepFFR workstation includes the neural networks set on each point of the vascular path. Structural and functional features of each point on the vascular centerlines are considered as input, while calculating FFR of each point as output. Therefore, DeepFFR is on the coronary tree simultaneously at a quick time at post-processing (Fig. 2). Lesion-specific CT-FFR is defined as simulated FFR value at distance of 20 mm away from the lesion of interest.

### Treatment arms

If the subjects are randomly allocated to CCTA/CT-FFR arm, they will be examined by DeepFFR for three major epicardial arteries. If the result of CT-FFR calculation is less than or equal to 0.8 in one or more major coronary arteries, the patient will be referred to ICA directly; if the result of CT-FFR value is more than 0.8, optimal medical therapy will be recommended. The decision on the mode of revascularization is left to the treating cardiologists and depends on local practice.

Correspondingly, if the subjects are randomized to usual care arm, attending physicians will decide the next step of diagnosis and treatment, such as exercise ECG, stress cardiac echo, cardiac MR, and SPECT. According to the results of examination combined with risk factors assessment and clinical manifestations, physicians should provide recommendation whether the subjects would undergo ICA or not.

The evaluation criteria of functional examination include but not all:

- The exercise ECG criterion for a positive test is greater ≥ 1 mm of horizontal or downsloping ST segment deviation (depression or elevation) in any lead except aVR for at least 60 to 80 ms after the end of the QRS complex, either during or after exercise;
- The nuclear cardiology criterion for a positive result is evaluated as follows: perfusion is graded using a 5-point scale (0 to 4) in each of 20 myocardial segments. Summed rest scores, summed stress scores, and summed difference scores (SDS) are recorded. Reversible defects are graded as small if SDS was 2 to 4, moderate if SDS was 5 to 8, or large if SDS was > 8. Subjects are categorized as having ischemia if more than 1 of the following criteria was present: SDS was ≥ 2 and/or there was an ungated stress-and-rest volume (transitory ischemic dilation) ratio of > 1.19;
- The stress cardiac echo criterion for a positive result is evaluated as follows: abnormal findings include those with fixed wall-motion abnormalities or new or worsening abnormalities indicative of ischemia. A segment with resting dysfunction may show either a sustained improvement during stress indicating a non-jeopardized myocardium (stunned) or improve during early stress with subsequent deterioration at peak (biphasic response). The biphasic response is suggestive of viability and ischemia, with jeopardized myocardium fed by a critically coronary stenosis. Resting wall motion abnormalities, unchanged with stress, are classified as “fixed” and most often represent regions of prior infarction.

The DICOM imaging data will be transferred to on-site workstation to complete DeepFFR measurement and the on-site lab will provide the report to the referral physician within 24 h for decision making.

On the consent form, participants will be asked if they agree to use of their data should they choose to withdraw from the trial. Participants will also be asked for permission for the research team to share relevant data with people from the hospital taking part in the research, where relevant. This trial does not involve collecting biological specimens for storage.

### Downstream decision making

The results of the index test will be provided to the reference cardiologist of the patients’ institution who will make clinical decisions based on the integrated evaluation of patient clinical assessment and index test findings. The following downstream decision making will be recorded from study entry until the end of follow-up: [1] non-invasive diagnostic tests, including further stress testing (exercise or pharmacological stress), with detection of ischemia by ECG, myocardial perfusion, or wall motion abnormalities; [2] number of ICA and prevalence of non-obstructive CAD at ICA; and [3] goals of risk factors control by optimal medical therapy.

At baseline, 6 months, and 12 months, recommendations for therapy are made in line with guidelines published. The goal of anti-hypertensive therapy is to achieve a blood pressure of less than 140/90 mmHg. The choice of anti-hypertensive therapy will be left to the treating physician. The aim of anti-lipid therapy is to achieve levels of LDL < 1.9 mmol/l. In the first instance, statin therapy will be initiated and then increased with the addition of a second agent if necessary. In the case of diabetics with a raised blood sugar, the primary health care physician is asked to measure HbA1c and to ensure that the patients’ subsequent therapy is tailored to achieve a HbA1c of less than 6.5 mg/dl. Smokers are referred to the smoking cessation clinic.

Data monitoring committee is responsible for generation of allocation sequence, and the investigators of each sub-center are responsible for the enrollment of subjects. After randomization, participants in the CT-FFR group will be assigned to receive CT-FFR examination according to the clinical physicians. The investigators will not take part in the recommendation of usual care for participants. Implementing CT-FFR or usual care will not require alteration to usual care pathways (including use of any medication), and these will be permitted to continue for both trial arms.

### Follow-up

Subjects will be contacted regularly by trained interviewers at 90 days, 6 months, and 12 months post-enrollment for follow-up assessment until death, withdrawal, or end of the trial. All subjects are followed for a minimum of 12 months. An independent clinical event adjudication committee (CEC) reviewed all primary endpoint event and secondary endpoints in a blinded fashion. The decisions of CEC will be used to implement the final statistical analysis.

The data were collected, coded, and entered by the trial investigators, and the paper-based CRF form is sent to the trial office by investigators to ensure that the data would not be tampered. The researchers used double data entry and range checks for data value method to ensure the accuracy of the data. A clinical research organization (CRO) has been contracted to oversee the monitoring of all sites, establishing the eCRF and checking the completeness and consistency of the trial data. Adherence to this trial will be monitored via hospital information system. The follow-up examination results should be sent to the research site, and the examination results of the grade 3 hospital can be accepted to maintain the credibility.

### Endpoint of the study

The primary endpoint of the present trial is comparison between the two arms in the rate of planned ICA without significant obstructive CAD within 90 days. Significant obstructive CAD is defined as more than or equal to 70% of area stenosis by quantitative analysis in core lab or invasive FFR ≤ 0.8 if available during procedure.

The secondary endpoint will be the comparison between the two treatment arms in terms of MACE, quality of life, cumulative effect dose of radiation exposure, and overall medical cost during the follow-up at 1 year.

The Seattle Angina Questionnaire (SAQ) was used to assess the clinical effect and quality of life (QOL). We will also measure the cumulative radiation exposure dose (ED) over the entire study period by assessing the original average dose for each test performed during the follow-up. In case the ED for each test is not known, we will use the standard ED available for each test in the literature.

Major adverse cardiovascular events (MACE) will be defined as a combined endpoint of (a) hospitalization for unstable angina, (b) revascularization by PCI or CABG after 90 days, (c) non-fatal MI, and (d) cardiac death: any death because of immediate cardiac cause (e.g., MI, low-output failure, fatal arrhythmia) or vascular cause (e.g., cerebrovascular disease, pulmonary embolism, ruptured aortic aneurysm, dissecting aneurysm, or other vascular cause). Unwitnessed death and death of unknown cause will be classified as cardiovascular death. An independent clinical event adjudication committee will review the agreement between all events and the provided definitions.

Adverse event (AE) monitoring will begin when a participant has been randomized and will continue for 1 year. We will record AEs which are defined as serious or which are potentially related to the intervention according to CT-FFR result independently. Since we defined MACE as a secondary endpoint, the SAEs (including death, cardiac events, hospitalization for unstable angina pectoris) are parts of the study. There is no anticipated harm and compensation for trial participation.

### Sample size calculation

The sample size is defined based on the rate of planned ICA without significant obstructive CAD within 90 days. Based on previous data and assuming the prevalence of non-obstructive CAD during ICA in usual care group is about 30% [7]. The frequency of reduction in the primary endpoint is expected to be 30% for a ≥ 90% power. Considering a drop-off up to 10%, the final overall population should be of 1216 patients.

### Statistical analysis

All data statistical analysis will be performed using Stata version 15.0 (StataCorp, College Station, Texas). The distributions [mean ± standard deviation (SD)] of the parameters are calculated. The parameters are compared between the groups using either Student’s *t* test for paired or Wilcoxon test for non-paired samples. The chi-square test is applied for the comparison of categorical variables. Multivariable regression or logistic regression is used for analysis of association of various parameters. Cox multivariable regression model is used to find the causal inference between clinical pathway and accordingly endpoints. The hazard ratio (HR) is presented as 95% confidence intervals (CI). *P* < 0.05 is considered as significance in statistics. Because there are no anticipated problems that are detrimental to the participants, interim analyses and formal stopping procedure are not necessary for this trial. Missing values will be managed by using multiple imputation. If there is any non-adherence with the trial protocol and intervention plan, investigators will record it truthfully. TSC will confirm whether there is a major protocol deviation and whether it can be used for data analysis. Intention-to-treat analysis will be applied for patients who do not adhere to the intervention. Based on the data of the current study, further subgroup analysis will be conducted for the additional purpose in the future.

### Date management and organization

In order to ensure and monitor the progress of TARGET registry trial, a Trial Steering Committee (TSC) has been established including the authors of this study. As the principal investigators, YC and JY are responsible for co-leading the study. They will ensure the integrity and standardization of the study by managing and supervising the study activities and report of the finding as a whole. They will facilitate closely with the sub-center, by initiating and maintaining communication among the study staff of six sub-center, meeting with the faculty investigators every month, and providing continuous supervision and support. DS, ZW, MD, XM, XH, and HZ, as the investigators of sub-center, are mainly responsible for identifying potential recruitment and taking consensus. One data collector will be based at each sub-center and will be responsible for recruiting participants and obtaining data through regular interviews. The data management team, led by JY, will be responsible for the storage, analysis, and interpretation of quantitative data. The team will clean up the data and code measures at each point in time to ensure that the data is valid and easy to be interpreted. The sponsor played no part in study design; collection, management, analysis, and interpretation of data; writing of the report; and the decision to submit the report for publication.

Data collected during the course of the research will be kept strictly confidential and only accessed by members of the trial team (or individuals from the sponsor organization or center sites where relevant to the trial). On the consent form, participants will be asked if they agree to use of their data should they choose to withdraw from the trial. Participants will also be asked for permission for the research team to share relevant data with people from the hospital taking part in the research, where relevant. This trial does not involve collecting biological specimens for storage. At present, there is no plan to share the data with other teams or organizations. The datasets analyzed during the current study are available from the corresponding author on reasonable request.

Results will be disseminated via a peer-reviewed report to the sponsor, which will be freely available, and through open access journal articles and conference presentations. Standard journal authorship criteria will apply; there will be no use of professional writers.

Informed consent forms are available from the corresponding author on request. If it is necessary to amend protocol, we will notify the sponsor and funder first then the primary investigator will notify the centers, and a copy of the revised protocol will be sent to the primary investigator to add to the Investigator Site File. Any deviations from the protocol will be fully documented using a breach report form, and the amendment of protocol will be updated in the clinical trial registry.

### Ethics statement

The study protocol is complied with the World Medical Association Declaration of Helsinki. Ethical clearance for the TARGET trial has been obtained from the ethical committee of Chinese PLA general hospital.

## Discussion

**Fig. 3:**
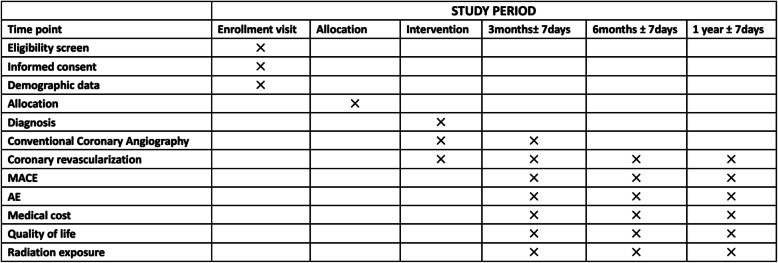
Chronology of the research (Standard Protocol Items: Recommendations for Interventional Trials (SPIRIT) Figure)

The core goal of TARGET trial is to assess the effect of CT-FFR on clinical decision making to the patients with stable chest pain in comparison of standard of care group (Fig. 3). The hypothesis is that CT-FFR-guided clinical management may provide extra benefit in reducing the rate of planned ICA without obstructive CAD, decreasing patients’ medical expenditure, and improving outcomes. This randomized control trial will help physicians to understand deeply the availability of CT-FFR as a non-invasive diagnostic method in the evaluation of myocardial ischemia.

Previous large cohort studies have confirmed the inconsistency between anatomical stenosis found by ICA and functional myocardial ischemia [11–13]. Due to the limitation of diagnosis based on CCTA imaging, it remains challenging to rely on anatomical evaluation solely for clinical management of patients. Although FFR measurement can be used to evaluate patients with stable chest pain during ICA, the risk of invasive procedure still exists. Moreover, some studies have demonstrated that less than half of patients with obstructive CAD were found by invasive angiography [14]. A study simultaneously evaluating anatomical and functional abnormality (COURAGE study) has found that only 32% of patients with severe coronary stenosis showed severe myocardial ischemia, while 40% showed no signs of ischemia or only mild ischemia [15]. These findings reflected the discrepancy between anatomical stenosis and myocardial ischemia, and functional assessment will be imperative for clinical management for the patients with stable chest pain.

Recently, CT-FFR technology brings out a new hope for anatomical and functional assessment in accuracy, simultaneously. The RIPCORD study has shown that CCTA combined with CT-FFR may lead to more caution for both ICA and followed by PCI when treating patients with stable angina pectoris [16]. Therefore, this combined strategy implies important clinical reference in selection of examination, clinical decision making, improvement of prognosis, and reduction of expenditure. The TARGET trial can better optimize the clinical management and improve the prognosis in a careful, strict, and randomized controlled design. The prospective data derived from the TARGET trial may assist us to answer this important question and provide more useful information.

Diagnostic performance of DeepFFR technique has been confirmed as previously described [10]. By learning corresponding invasive FFR values of coronary lesions from a large number of existing databases, this AI-based technology could generate neural network model by deep learning algorithm. The major time consumption depends on the recognition of the centerline of the coronary artery and manual correction of boundary. The calculating time has been dramatically reduced, so the waiting time for patients is greatly shortened. However, previous CT-FFR strategy relies on the transfer of imaging data into the cloud or the core lad, which may increase time consumption and even induce the “black-box” effect. For on-site measurement, CT-FFR value can feedback to physician within 1 day, which is conducive to rapid decision making downstream. On the other hand, clinical management based on DeepFFR may greatly reduce expenditure, may save the cost of diagnosis and treatment, and will be more easily taken into practice in developing countries.

In conclusion, the purpose of the TARGET trial is to evaluate whether clinical decisions based on CT-FFR measurements could decrease unnecessary ICA, optimize diagnostic and therapeutic procedures, reduce radiation exposure, save medical expenditure, and improve prognosis in comparison of conventional management for patients with stable chest pain. The effect will be assessed by the rate of non-obstructive CAD in planned ICA within 90 days and MACE within 12 months. Additionally, the impact includes QOL, reduction of cumulative radiation exposures, and medical expenditure within 12 months because reduction of overuse of invasive procedure will be evaluated as well. In brief, the TARGET trial aims to provide a new concept on health care for the management of suspect CAD patients in China.

## Trial status

The current protocol is version 2.0 (201812) and was issued on 1 January 2018. Recruitment of patients and data collection started in August 2019. Recruitment of patients will be finished in December 2020. The 1-year follow-up will be completed in December 2021.

## Supplementary information

**Additional file 1.** SPIRIT 2013 Checklist: Recommended items to address in a clinical trial protocol and related documents.

## Abbreviations

CCTA: Coronary CT angiography
CAD: Coronary artery disease
ICA: Invasive coronary angiography
FFR: Fractional flow reserve
PCI: Percutaneous coronary intervention
CI: Confidence interval
AI: Artificial intelligence
MACE: Major adverse cardiac event
HR: Hazard ratio
SD: Standard deviation
ED: Exposure dose
